# Albumin-binding photosensitizer capable of targeting glioma via the SPARC pathway

**DOI:** 10.1186/s40824-023-00360-3

**Published:** 2023-03-21

**Authors:** Xingshu Li, Jae Sang Oh, Yoonji Lee, Eun Chae Lee, Mengyao Yang, Nahyun Kwon, Tae Won Ha, Dong-Yong Hong, Yena Song, Hyun Kyu Kim, Byung Hoo Song, Sun Choi, Man Ryul Lee, Juyoung Yoon

**Affiliations:** 1 Fujian Provincial Key Laboratory for Cancer Metastasis Chemoprevention and Chemotherapy, College of Chemistry, Fuzhou University, Fuzhou, China; 2 Department of Neurosurgery, College of Medicine, Cheonan Hospital, Soonchunhyang University, Cheonan-si, Chungcheongnam-do Republic of Korea; 3 Department of Neurosurgery, Uijeonbu St. Mary’s Hospital, College of Medicine, The Catholic University of Korea, Seoul, Republic of Korea; 4 College of Pharmacy, Chung-Ang University, Seoul, Republic of Korea; 5 Department of Chemistry and Nanoscience, Ewha Womans University, Seoul, Republic of Korea; 6 Soonchunhyang Institute of Medi-bio Science (SIMS), Soonchunhyang University, Cheonan-si, Chungcheongnam-do Republic of Korea; 7 Global AI Drug Discovery Center, College of Pharmacy and Graduate School of Pharmaceutical Sciences, Ewha Womans University, Seoul, Republic of Korea

**Keywords:** Glioma, Blood-brain-barrier, Photosensitizer, Albumin binding, Secreted protein acidic and rich in cysteine

## Abstract

**Background:**

Malignant glioma is among the most lethal and frequently occurring brain tumors, and the average survival period is 15 months. Existing chemotherapy has low tolerance and low blood-brain barrier (BBB) permeability; therefore, the required drug dose cannot be accurately delivered to the tumor site, resulting in an insufficient drug effect.

**Methods:**

Herein, we demonstrate a precision photodynamic tumor therapy using a photosensitizer (ZnPcS) capable of binding to albumin in situ, which can increase the permeability of the BBB and accurately target glioma. Albumin-binding ZnPcS was designed to pass through the BBB and bind to secreted protein acidic and rich in cysteine (SPARC), which is abundant in the glioma plasma membrane.

**Results:**

When the upper part of a mouse brain was irradiated using a laser (0.2 W cm^− 2^) after transplantation of glioma and injection of ZnPcS, tumor growth was inhibited by approximately 83.6%, and the 50% survival rate of the treatment group increased by 14 days compared to the control group. In glioma with knockout SPARC, the amount of ZnPcS entering the glioma was reduced by 63.1%, indicating that it can target glioma through the SPARC pathway.

**Conclusion:**

This study showed that the use of albumin-binding photosensitizers is promising for the treatment of malignant gliomas.

**Graphical Abstract:**

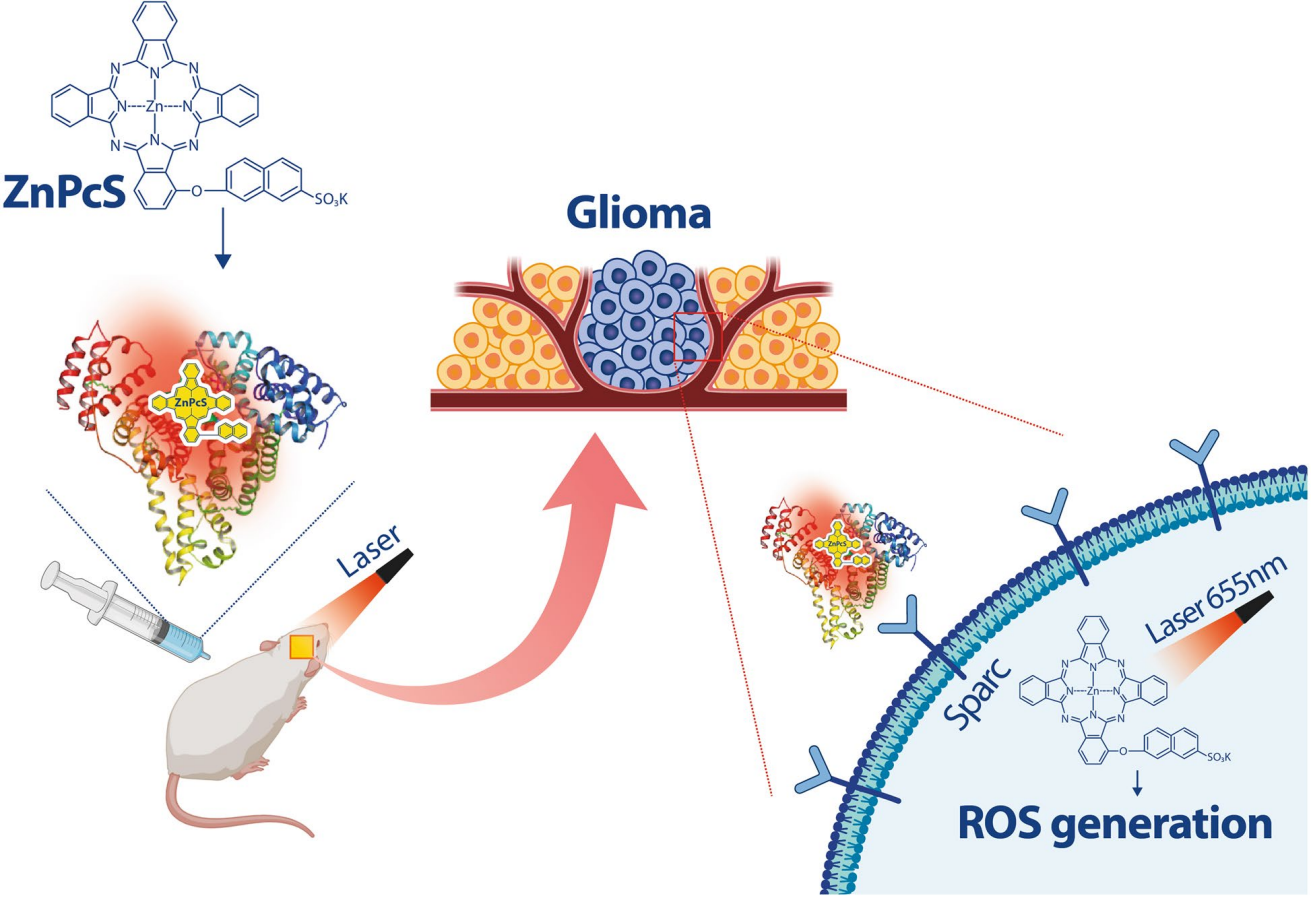

**Supplementary Information:**

The online version contains supplementary material available at 10.1186/s40824-023-00360-3.

## Background

Malignant gliomas, which occur more frequently than other types of primary central nervous system tumors, are the most aggressive type of tumors [1]. Various treatments have been developed over the past 20 years, including chemotherapy, radiotherapy, photodynamic therapy [2], hyperthermia therapy, and immunotherapy [3–6], to improve the quality of life of patients by completely eliminating glioma and prolong patient survival. Currently, the most popular glioma treatment method is the co-administration of temozolomide (TMZ) after surgery [7]. However, due to the heterogeneity and diversity of glioma and the unique tumor microenvironment, existing treatment methods cannot achieve a significant clinical effect, leading to a five-year overall survival rate of only 35% [3, 8–10]. Existing treatments are inefficient and mortality is high owing to the difficulty of completely surgically excising glioma cells that have locally invaded the brain [11, 12]. In addition, brain vessels have a blood-brain barrier (BBB), which is a physical and chemical barrier that prevents existing anticancer drugs from accessing glioma successfully, making treating glioma more challenging than other tumors [13, 14]. The BBB surrounding cerebral blood vessels is composed of a continuous endothelial monolayer connected by dense, tight junctions. Its role is to significantly reduce the permeability for material exchange, thereby preventing the movement of inflammatory cells or brain damage. However, the BBB acts as a barrier to the delivery of drugs to brain cells. To maximize BBB permeability, a method of temporarily destroying the BBB with ultrasound and thereby improving drug delivery has been frequently used in recent years; however, the destruction of the BBB poses a threat of side effects [15]. To overcome the limitations of existing treatments, a therapeutic approach using glioma-specific membrane proteins could be adopted to achieve homogeneous, effective, and accurate drug treatment for deep solid tumor gliomas by increasing BBB permeability. In this regard, the use of nanoparticles for safe and effective delivery and precise targeting of drugs to the brain without opening or damaging the physical BBB has been presented.

Even if nanoparticles pass through the BBB without causing physical damage to it, a second gateway is required for precise targeting of the glioma region to maximize the anticancer effect of nanoparticles and protect the surrounding tissues. Because tumor cells, including gliomas, proliferate abnormally fast, they consume a significant amount of energy to maintain this growth process and require many nutrients, such as albumin [16]. Although albumin is generally restricted from entering the brain, the rapid growth of highly metabolically active tumors causes them to become starved for nutrients, and tumor cells significantly enhance albumin uptake into glioma tissues for use as an amino acid and energy source. To increase the absorption of albumin into glioma, secreted protein acidic and rich in cysteine (SPARC), a type of albumin-binding protein, is maintained in an overexpressed state compared to the surrounding vascular endothelial cells and nerve cells, and albumin is introduced into glioma in large quantities through the endocytosis of tumor cells [17]. SPARC is an extracellular glycoprotein that is abnormally overexpressed in solid cancers, including brain tumors, and acts as a promoter of tumor proliferation and metastasis. Furthermore, SPARC is expressed at low levels in a normal adult brain but is overexpressed in all grades of astrocytoma, particularly in regions where metastasis is active [15]. Therefore, we believe that the SPARC-mediated glioma targeting strategy could be effective for the precise and selective accumulation of drugs in gliomas to safely pass through the BBB and effectively deliver drugs to the target region.

Generally, nanotheranostic systems containing specific targeting peptides, aptamers, or other ligands have been used as an effective approach for cancer treatment [18–20]. Non-invasive all-in-one theranostic nanoparticles that can accurately deliver an appropriate therapeutic agent to a target for effective local control of tumors are emerging as a new strategy for targeted tumor treatment, including glioma treatment [21, 22]. For nanoparticles to effectively suppress tumor cells without drug resistance, they must contain a functional group that can participate in a chemical reaction as a catalyst [3, 23]. Various functional groups can be attached to synthetic nanoparticles. In this study, α-(6-sulfonatonaphthaleneoxyl) phthalocyaninato zinc (ZnPcS) was synthesized to kill gliomas by producing highly reactive oxygen species (ROS) under appropriate light irradiation. ZnPcS was applied to directly kill tumor cells by generating short-lived ROS upon light irradiation, operating only in cells within a few tens of nanometers. In particular, ZnPcS with an amphiphilic structure was used to target gliomas overexpressing SPARC by strongly binding to albumin in situ. We determined whether the synthesized ZnPcS could be injected intraperitoneally to pass through the BBB and accurately suppress glioma and whether ZnPcS accurately targets glioma through SPARC in vivo and in vitro. The results of this study will be useful for evaluating the efficacy of albumin-binding ZnPcS as a new photosensitizer for glioma treatment in preclinical animal models.

## Methods

### Chemical materials and instruments

Potassium 6-hydroxy-2-naphthalenesulfonate, phthalonitrile, 3-nitrophthalonitrile, potassium carbonate, zinc acetate, dimethyl sulfoxide (DMSO), n-pentanol, 1,8-diazabicyclo-[5.4.0]undec-7-ene (DBU), and 2,7-dichlorofluorescin diacetate (DCFH-DA) were purchased from Sigma-Aldrich, Korea. High-resolution mass spectra were obtained using Exactive Plus Orbitrap (Thermo Fisher Scientific). Proton nuclear magnetic resonance spectra were recorded on a Bruker 300 MHz. Fluorescence spectra were collected using an Edinburgh FL900/FS900 spectrofluorometer. Electronic absorption spectra were recorded using a SHIMADZU UV-2450 spectrophotometer.

### Molecular modeling studies

####  Protein and ligand preparation

The X-ray crystal structures of human serum albumin (HSA) bound to heme molecules (PDB codes:1N5U3 and 1O9 × 4) were prepared using the Protein Preparation Wizard in Maestro, version 9.2 (Schrödinger, LLC, NY, USA) [24, 25]. Bond orders were assigned, hydrogen atoms were added, and protonation states of the residues at pH 7.4 were generated by Epik, version 2.6. All hydrogen atoms were energy-minimized with the optimized potential for liquid simulation (OPLS) 2005 force field until the average root-mean-square deviation in the hydrogen atoms reached 0.30 Å. Metal coordination in the ZnPcS ligand was prepared using the protein preparation wizard. The resulting structures were energy-minimized using the implicit solvent model and OPLS2005 force field.

#### Blind docking and binding mode selection

The ligands were docked into several binding pockets of HSA using Glide, version 6.1, in Maestro, according to the following protocol: (i) The grid box was generated using the centroid of the co-crystallized ligand. To ensure that all the binding cavities in the HSA were covered, we set the grid box size to be sufficiently large. (ii) Glide standard precision docking was used for the docking stage, and up to 500 conformations of each ligand were identified. (iii) The docked conformations were clustered based on their root-mean-square deviations, and the final modes were chosen based on their scores and population.

The computational tasks were performed on a Linux CentOS 5.8 workstation with an Intel Xeon octa-core 2.5 GHz processor, and molecular graphic figures were generated using PyMOL software (http://www.pymol.org).

### Cell culture

The human glioma U87 cell line was purchased from the American Type Culture Collection (ATCC, USA) and cultured in Eagle’s Minimum Essential Medium with 1.5 g L^− 1^ sodium bicarbonate, nonessential amino acids, L-glutamine, sodium pyruvate, and 1% Penicillin/Streptomycin (Corning, NY, USA) with 10% heat-inactivated fetal bovine serum (FBS, GWVITEK, Seoul, Korea). The human umbilical vein endothelial cell (HUVEC) cell line was purchased from ATCC and cultured in EGM2 medium with supplements. Confluent cells were passaged every 4–5 days using 0.05% trypsin/ethylenediaminetetraacetic acid. All the cells were maintained in an incubator at 37 °C with 5% CO_2_ in a humidified atmosphere.

### Immunocytochemistry

To demonstrate the accumulation of ZnPcS in glioma cells, immunocytochemistry was performed using confocal microscopy (LSM710; Carl Zeiss Microimaging GmbH, Jena, Germany). Briefly, the cells were cultured in a 35 mm confocal microscope dish for 48 h and then treated with ZnPcS at the indicated concentrations. After the indicated incubation times, the cells were first washed in PBS and then fixed with 4% paraformaldehyde for 20 min. After a series of washes with PBS, the 4’,6-diamidino-2-phenylindole (DAPI) (Thermo Fisher Scientific, Waltham, MA, USA) was treated to make the nuclear contrast. To identify the protein expression of SPARC, the SPARC antibody was applied to fixed cells. After washing twice with PBS, the samples were blocked with 5% bovine serum albumin (BSA) for 1 h at room temperature and washed again with PBS. The cells were incubated with the primary antibody overnight. The dishes were washed thrice with PBS/0.05% Triton X-100 and incubated with the secondary antibody for 2 h at room temperature in the dark. After washing with PBS/Triton X-100, the cells were counterstained with DAPI. All the fluorescence images were captured using a Carl Zeiss confocal laser scanning microscope with a 20× objective.

### Photo-induced cytotoxicity against U87-glioma

The effect of ZnPcS on U87-glioma viability was measured using the MTT assay. Briefly, 1 × 10^4^ cells were seeded in 96-well tissue culture plates. After 24-h incubation, the cells were treated with different concentrations of ZnPcS (0–20 µM in MEM medium) for 24 h. The cells were recharged with fresh medium, exposed to laser irradiation (655 nm, 200 mW, 1 min), and further incubated for 24 h. Cell viability was evaluated using the MTT assay (Theranostics 2019). MTT reagent was added to each well, and the cells were incubated for 4 h. The DMSO was added to dissolve the formazan crystals, and the absorbance of the purple formazan was measured at 570 nm using a microplate reader.

### Intracellular ROS detection

ROS generation within the cells was identified using DCFH-DA as a ROS indicator by means of fluorescence. The cell-permeable non-fluorescent DCFH-DA can freely cross the cell membrane to enter the cell and can be oxidized to form a green fluorescent DCF. To identify ROS generation in culturing U87-glioma, the cells were cultured in a confocal culture dish. After one hour of ZnPcS treatment of U87, the cells were subjected to laser irradiation for 1 min. The cells were washed thrice, and the culture medium of DCFH-DA (10 µM) was further incubated with the cells for 30 min (biomacromolecules).

#### Penetration of ZnPcS using in vitro BBB model

To determine whether ZnPcS can penetrate the BBB, an in vitro BBB model using a Transwell was used. The human endothelial cell line HUVEC was seeded in a Transwell upper chamber (Corning, USA) for monolayer cell culture. The cells were cultured for two days to become fully confluent. The U87-glioma was seeded in the lower chamber. The upper chamber was treated with ZnPcS, and the U87-glioma cells in the lower chamber were collected and washed with PBS to obtain fluorescence images.

### In vivo of intracranial glioma xenograft model

Five-week-old female Balb/c nude mice were purchased from Nara Biotech (Seoul, Korea), and one week later, the experiment was started with six-week-old mice under specific pathogen-free conditions. To briefly explain the entire process, orthotopic glioma tumor mouse models were developed by transplanting Luc-U87 (stable transfection of firefly luciferase) into the left cerebral hemisphere using a stereotaxic apparatus (Harvard, MA, USA). The skull was leveled between the bregma and lambda. A small hole was drilled at the desired location and 1 × 10^6^ Luc-U87M cells in 3 µL of PBS were injected into the frontal lobe based on the following coordinates (relative to Bregma) using a Hamilton syringe (10 µL Gastight Syringe Model, 1701 RN, Small Removable Needle, 26s gauge, 2 in, point style 2, Reno, Hamilton Company, NV, USA, Nevada, USA): 2.7 mm ventral from the dorsal surface of the skull, 0.5 mm caudal, and 1.1 mm lateral. The rate at which cells enter the brain was set to 0.5 µL/min, and the cells were injected. The growth of orthotopic glioma was monitored using bioluminescence imaging performed in the IVIS imaging system by intraperitoneal injection of luciferin substrate (150 mg/kg) [17].

To follow the ZnPcS distribution in vivo, fluorescence was measured in the major tissues after the IP administration of ZnPcS. After treatment with ZnPcS, the mice were euthanized to collect major tissues, including the brain, for fluorescence imaging at 1 h. The mice had continuous access to sterilized food pellets and distilled water and were housed under a 12 h light-to-dark cycle (7:00 am–7:00 pm) with a temperature of 23 °C ± 1–2, and humidity of 50%±5. All the animal experiments were approved by the Institutional Animal Care and Use Committee of Soon Chun Hyang University and performed in accordance with the guidelines of the Experimental Animal Center of Soonchunhyang Institute of Medi-Bio Science.

### In vivo antiglioma therapy

Mice with intracranial gliomas were randomly divided into five groups. The growth of orthotopic gliomas was monitored using luciferin for in vivo imaging. Treatment with ZnPcS was conducted seven days after Luc-U87 implantation, and the brain was irradiated by a 600 J laser. After irradiation, the growth of the orthotopic glioma was measured by the intensity of fluorescence, and the glioma size was monitored every week using IVIS. The survival rate and median survival time were calculated, and statistical differences were assessed using the Kaplan–Meier method.

### Image analysis

Anesthetized mice were placed in a light-tight chamber of Living Image version 4.5 (PerkinElmer, USA). For in vivo imaging, the mice were anesthetized with isoflurane (Hana Pharm, South Korea). Luciferase expression at the tumor site was visualized with the injection of luciferin (XenolightTM D-Luciferin Potassium Salt, 1 g, PerkinElmer, Waltham, MA, USA) and imaged by IVIS, and the luminescence intensity represented the tumor progression in the brain.

The mice were euthanized under isoflurane anesthesia prior to tissue collection via thoracotomy for biodistribution. Pseudo-color images indicating photon counts were overlaid on the images of the mice using Living Image software v. 2.25 (Caliper). A region of interest was manually selected based on the signal intensity. The area of the region of interest was kept constant, and the intensity was recorded as the maximum radiance within each region of interest [26].

#### SPARC knock-out

The CRISPR/CAS9 SPARC Knockout Kit (ORIGENE, KN409964) was used for SPARC knockout. The SPARC gRNA CRISPR vector (KN409964G1, KN409964G2) and non-homology-mediated-based linear donor DNA (KN409964D, EF1a-Luciferase-P2A-Puro) were transfected by nucleofection. Transfections were performed using the Amaxa NucleofectorTM system (SE Cell Line 4D-NucleofectorTM X Kit L, LONZA), according to the supplier’s recommendations. For each cell, a control transfection with the pmax-GFP (provided with the Amaxa Nucleofector kit) plasmid alone, and co-transfection with the pmax-GFP plasmid along with the SPARC gRNA CRISPR plasmid and donor DNA, was performed. For each transfection, cells were resuspended in 100 µL nucleofector solution, 1 µg of each plasmid and donor DNA was added, and electroporation settings (program: DS126, CA137) were applied and then seeded in six-well plates with 2 ml growth media. Transfected cells were grown in growth media for 11–48 h until the majority of the cells showed visible GFP expression. After sufficient growth, SPARC-knockout cells were selected using puromycin (Gibco).

### Western blotting

Whole cell lysates were extracted using NP40 (Elpis Biotech, Daejeon, Korea) with a 100× protease inhibitor cocktail (Cell Signaling Technology, Danvers, MA, USA). The total protein concentration in each sample was measured using the Bradford assay. The protein samples were separated by electrophoresis using 12% SDS-polyacrylamide gels and transferred to 0.2-µM polyvinylidene fluoride blotting membranes (Amersham, Little Chalfont, UK). The membranes were blocked for 1 h in TBS-T (50 mM Tris, 0.15 M sodium chloride, 0.05% Tween 20) containing 5% blocker (BioShop, Burlington, Canada) and hybridized to primary antibody overnight at 4 °C. The primary antibodies used were SPARC (Santa Cruz Biotechnology, Dallas, TX, USA) and β-actin. The membranes were washed thrice for 15 min with TBS-T at room temperature and incubated for < 1.5 h at room temperature with secondary antibodies. Chemiluminescence detection was performed using the Pico EPD Western Blot Detection Kit (Elpis Biotech) [27].

## Results

**Fig. 1:**
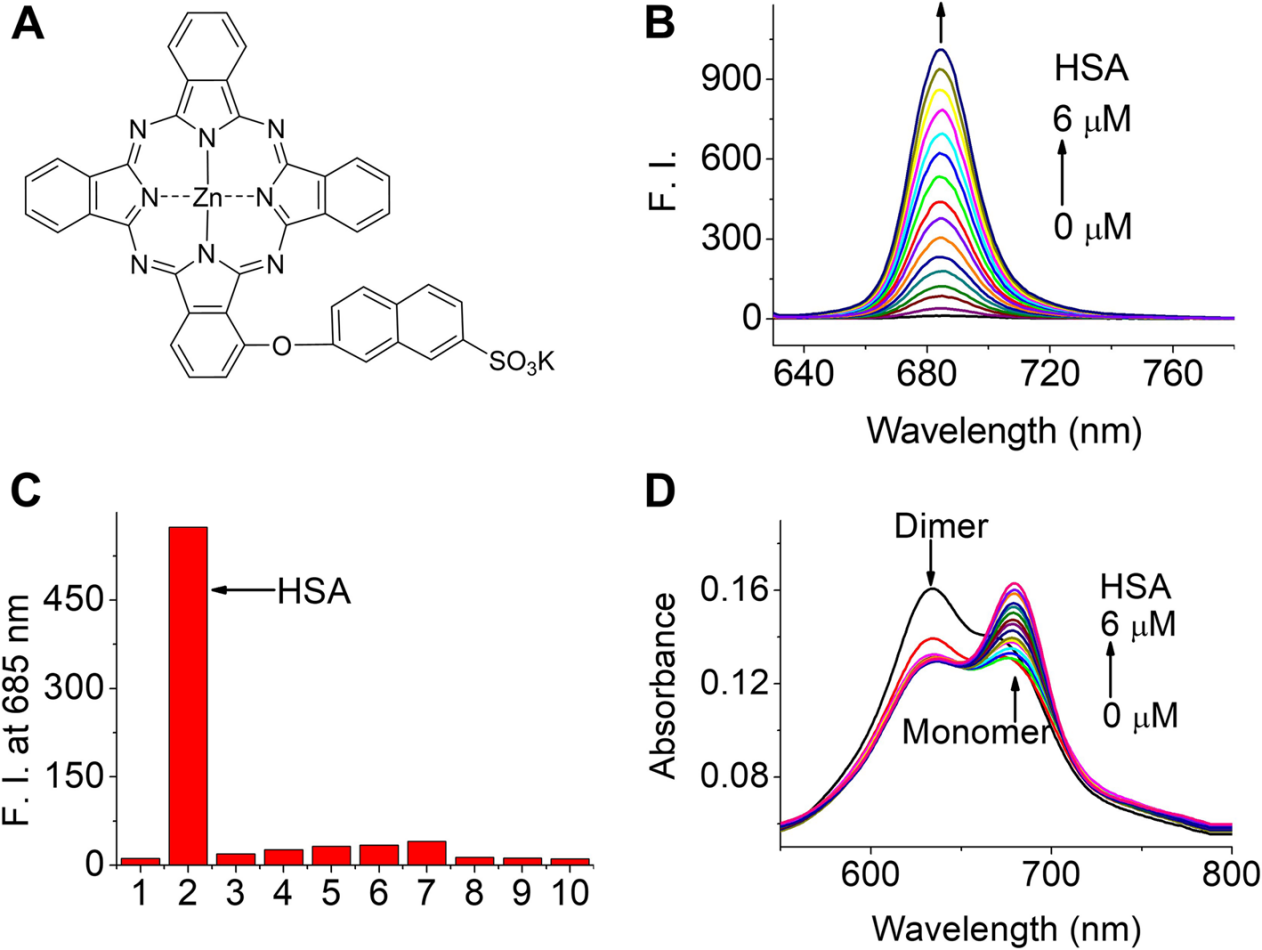
Structural and optical properties of ZnPcS. **a** Chemical structure of ZnPcS. **b** Fluorescence spectrum (excited at 610 nm) of ZnPcS (3 µM) in water with different HSA concentrations. **c** Fluorescence intensity of ZnPcS (3 µM) co-incubated with different types of proteins (all at 3 µM) and nucleic acids (30 µg/mL): 1 Control. 2 HSA. 3 Transferrin. 4 Protease. 5 Trypsin. 6 Lysozyme. 7 Fibrinogen. 8 IgG. 9 DNA. 10 RNA. **d** Absorption spectrum of ZnPcS (3 µM) in water with different HAS concentrations

Figure 1A shows the chemical structure of ZnPcS. Its synthesis and characterization are presented in the Supplementary Information, Additional File 1. We analyzed the interaction between ZnPcS and HSA using fluorescence spectroscopy. As shown in Fig. 1B, ZnPcS shows increased fluorescence intensity at 685 nm with increasing HSA concentration. However, there was no change in the fluorescence intensity, even when different types of proteins and nucleic acids were used (Fig. 1C). This result shows that ZnPcS can emit fluorescence through selective binding with HSA. The increased absorption at approximately 675 nm (which indicates a monomer) and decreased absorption at approximately 635 nm of ZnPcS upon addition of HSA suggest that HSA could reduce the aggregation of ZnPcS in aqueous solutions (Fig. 1D).

**Fig. 2:**
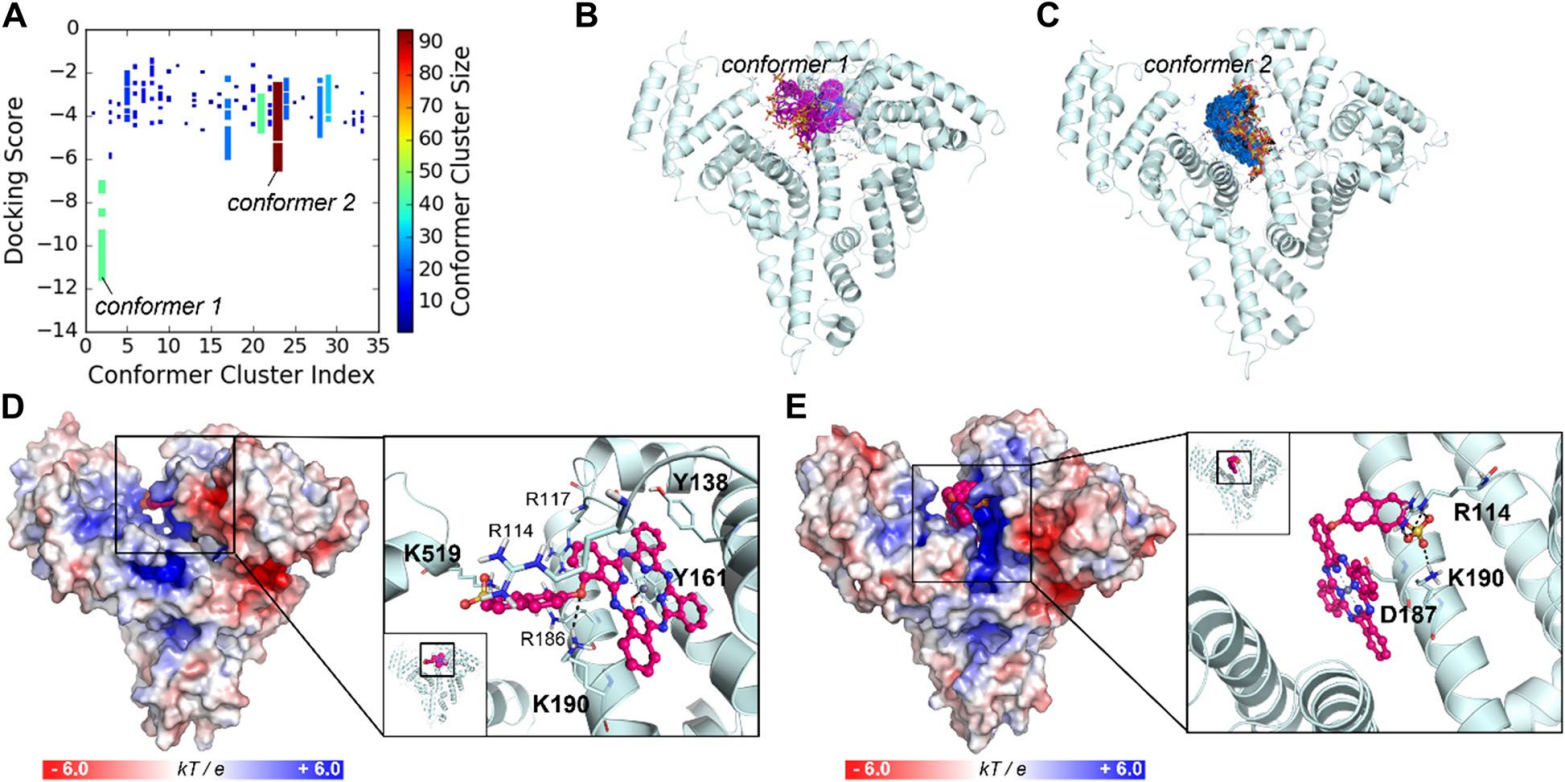
Predicted binding modes of ZnPcS at HSA. **a** Distribution of docking scores for conformer clusters. The 500 binding conformers of ZnPcS were clustered based on their root-mean-square deviations (RMSDs). The point color and size depend on the cluster size. The conformers with the lowest energy score and the most densely populated conformers are named *conformer 1* and *conformer 2*, respectively. **b** The conformers with the lowest energy scores are represented by sticks with their carbon atoms in purple. **c** The most highly populated conformers are displayed in sticks with their carbon atoms in sky blue. **d** The binding mode of ZnPcS at the heme site (*conformer 1*) of HSA. **e** The other possible binding mode of ZnPcS at the cleft region (*conformer 2*). The protein surface is colored according to its electrostatic potential, and the bound compound is represented by its carbon atoms in magenta. In the enlarged view, the compound is displayed by a ball-and-stick model, and the key residues that interact with the compounds are shown as thin sticks with their carbon atoms in light blue. The dative bond between the Zn^2+^ atom (shown in the purple sphere) and Y161 or D187 is indicated by black lines, and the ionic interactions are indicated by black dashed lines

To predict the binding mechanism of ZnPcS on HSA, we conducted molecular modeling studies. As shown in our earlier study on phthalocyanine nanovesicles, HSA has several fatty acid-binding sites (FA1-7), including the most well-known drug-binding sites near FA7 and the largest pocket where the heme molecule binds (FA1) [24, 25]. Owing to the structural similarity of phthalocyanines and the porphyrin group of heme, ZnPcS derivatives are speculated to be bound to the heme site. The other FA sites were too small for ZnPcS binding. Using blind docking with a grid that covers most of the binding cavities in HSA, we sampled 500 binding conformations per compound (Fig. 2A). Two distinct binding modes were observed in the docking study: one at the heme-binding site (Fig. 2B and D) and the other at the cleft region (Fig. 2C and E). This binding mechanism is similar to that of our previous study on phthalocyanine nanovesicles [28].

**Fig. 3:**
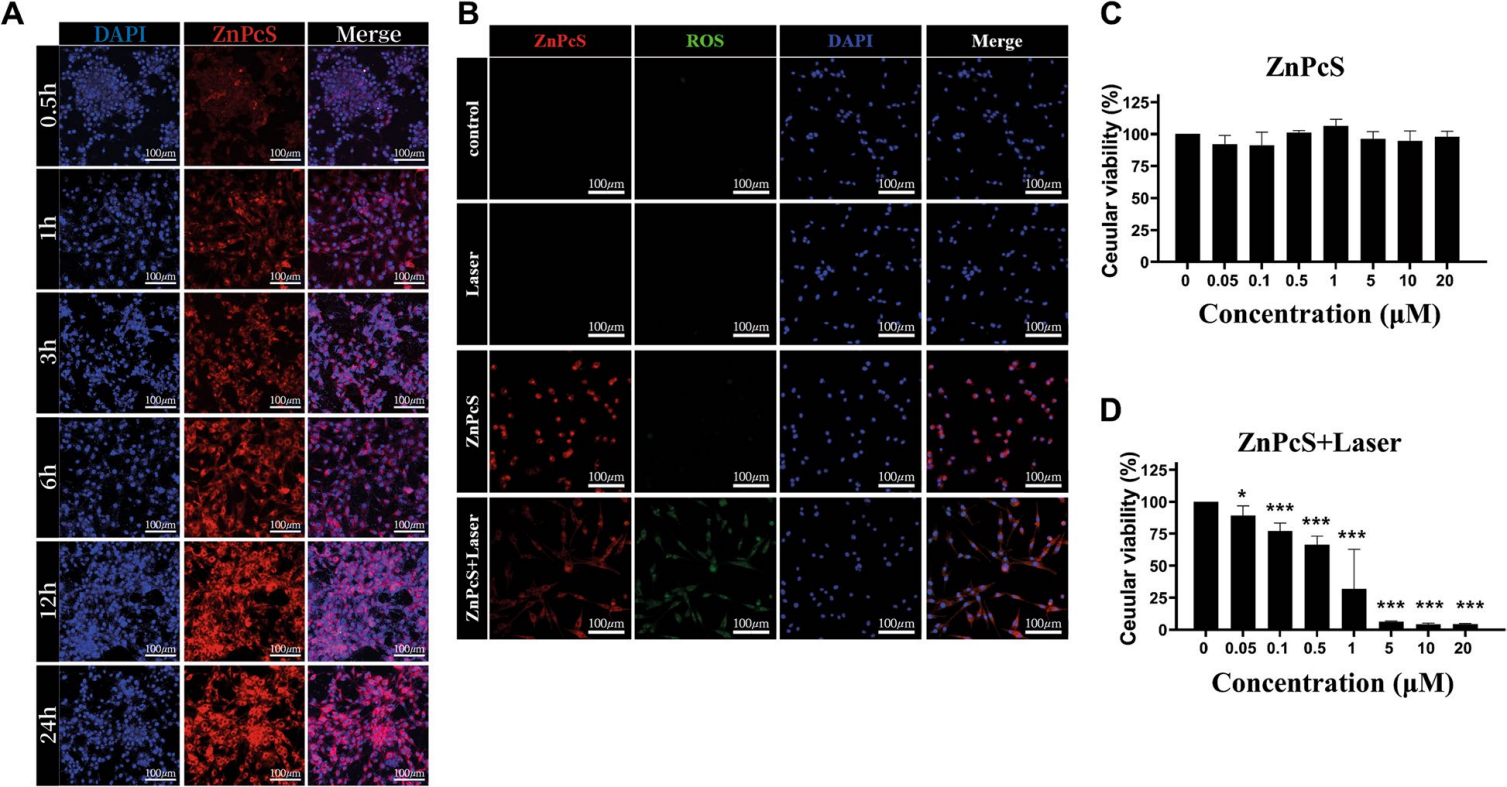
ZnPcS treated U87-glioma cells. **a** Cellular uptake of ZnPcS in U87-glioma over time; free porphyrin for 0.5, 1, 3, 6, 12, and 24 h (*n* = 3). The images from left to right show ZnPcS fluorescence (excitation 633 nm and emission 710 nm) and nuclear staining with DAPI (excitation 405 nm and emission 459 nm). Scale bar = 50 μm. **b** Intracellular ROS levels evaluation by DCF-DA (excitation 488 nm and emission 516 nm). Confocal microscopy images of U87-glioma after laser irradiation in ZnPcS treatment or nontreatment groups. Scale bar = 50 μm. **c**–**d** U87-glioma viability measured by the MTT assay after ZnPcS treatment with or without laser irradiation (*n* = 6). Quantitative data are represented as fold changes relative to the nontreated group and are displayed as mean ± SEM. Statistical analyses are performed by student t-test (*p < 0.05 and *** *p* < 0.001 compared with the nontreated group)

To demonstrate the uptake of ZnPcS into U87-glioma cells, we employed confocal microscopy. After treating 5 µM ZnPcS in the U87-glioma cell line in culture, we checked the fluorescence of ZnPcS after 0.5, 1, 3, 6, 12, and 24 h. Intracellular uptake began from the initial time (0.5 h) after ZnPcS treatment, red fluorescence appeared in the cytoplasm, and intracellular accumulation of ZnPcS increased over time. These results showed that 1 h of treatment with ZnPcS in cells was sufficient for ZnPcS accumulation in U87-glioma (Fig. 3A).

Effective ROS generation by ZnPcS upon 655 nm laser irradiation is important for glioma death. Thus, the capacity for intracellular ROS generation by ZnPcS in U87-glioma was measured by confocal microscopy using 2,7-dichlorofluorescin diacetate (DCF-DA) as the fluorescent probe. When only ZnPcS was treated or only laser irradiation was used, the ROS indicator (DCF-green fluorescence) was barely detected. However, when the ZnPcS-treated U87-glioma cells were irradiated by the laser (0.2 W cm^− 2^), the ROS indicator was observed throughout the cells. These results suggest that approximately no ROS were detected in the control cells and that ROS were generated from ZnPcS by laser irradiation (Fig. 3B).

MTT analysis and optical microscopy were used to determine whether ZnPcS alone exhibited cytotoxicity in the absence of laser irradiation (Fig. 3C). After loading 5 × 10^4^ cells per well in a 96-well plate, U87-glioma cells were treated with 0 µM to 20 µM of ZnPcS the next day. Even at a high concentration of 20 µM, roughly no apoptosis was observed. When the U87-glioma treated with each ZnPcS concentration was irradiated by the laser, cell death significantly increased depending on the ZnPcS concentration (Fig. 3D). These results showed that ZnPcS can rapidly penetrate glioma cells and ZnPcS itself has no cytotoxicity and can trigger cell death by ROS generation upon laser irradiation.

**Fig. 4:**
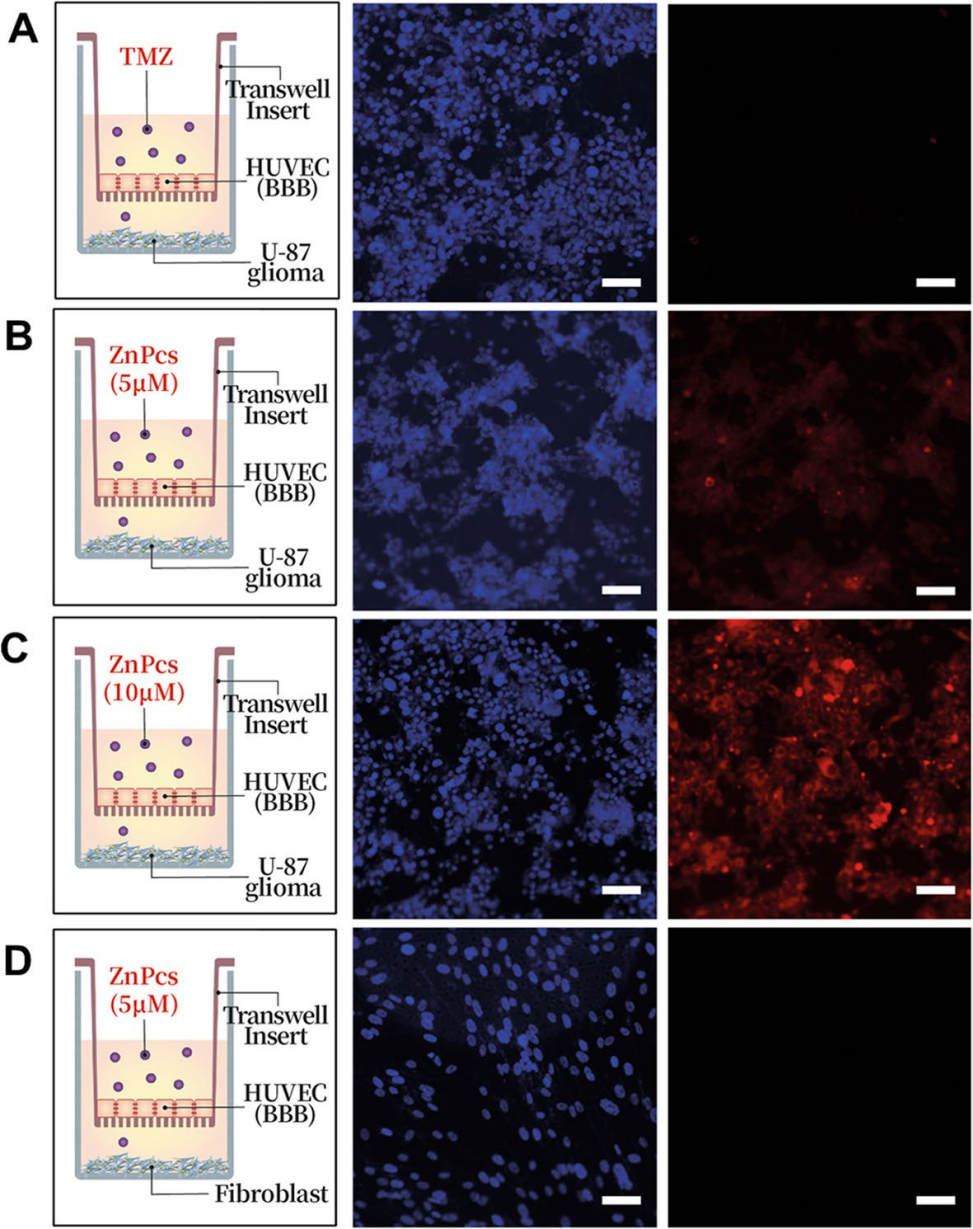
In vitro cellular mode for checking the ZnPcS passage across the BBB (*n* = 3). **a** HUVEC are cultured on a transwell membrane insert, and U87-glioma cells are cultured on the bottom of the well. Twenty-four hours after TMZ treatment, the U87-glioma cells are stained with DAPI and imaged. **b** HUVEC are cultured on a transwell membrane insert, and U87-glioma cells are cultured on the bottom of the well. Twenty-four hours after 5 µM ZnPcS treatment, U87-glioma cells are stained with DAPI and imaged. **c** HUVEC are cultured on the transwell membrane insert, and U87-glioma cells are cultured on the bottom of the well. Twenty-four hours after 10 µM ZnPcS treatment, U87-glioma cells are stained with DAPI and imaged. **d** HUVEC are cultured on a transwell membrane insert, and fibroblasts are cultured on the bottom of the well. Twenty-four hours after 5 µM ZnPc treatment, U87-glioma cells are stained with DAPI and imaged. In all the images, the blue fluorescent represents the nucleus, and the red fluorescent represents the ZnPcS. All the schematics show the sequence of cells in the transwells. ZnPcS (red fluorescence) is excited at 633 nm and monitored at 710 nm, and nucleus (blue fluorescence) is excited at 405 nm and monitored at 459 nm. Scale bar = 100 μm

To determine whether ZnPcS passes through the BBB to enter the glioma, an experiment was conducted using a Transwell. The monolayer culture in a Transwell using endothelial cells is a commonly used in vitro BBB model to study drugs targeting the brain [17, 27]. To simulate the BBB connected by a tight junction, HUVEC cells were sufficiently cultured in the upper chamber until confluence was achieved (Fig. 4A-D). To check whether ZnPcS was selectively introduced into the glioma cells, U87 cells (Fig. 4A-C) and a fibroblast cell line (BJ1: Fig. 4D) were loaded and cultured in the lower chamber. The upper well was treated with TMZ, the most commonly used anticancer agent for glioma treatment, to determine the status of glioma in the lower well (Fig. 4A). Although it is unclear whether TMZ easily passed through the in vitro BBB structure, no morphological changes or apoptosis of the glioma cells in the lower chamber occurred within 24 h after TMZ treatment. Furthermore, 5.0 µM ZnPcS (Fig. 4B) and 10.0 µM ZnPcS (Fig. 4C) were added to the upper chamber. The inflow of ZnPcS was confirmed in U87 cells in the lower chamber, and the inflow of ZnPcS increased in a concentration-dependent manner. The intracellular inflow of ZnPcS (5.0 µM) in the lower chamber fibroblast, however, was not observed. This suggests that ZnPcS can be selectively introduced into gliomas through the BBB, particularly through membrane proteins specifically present in the glioma cell membrane.

**Fig. 5:**
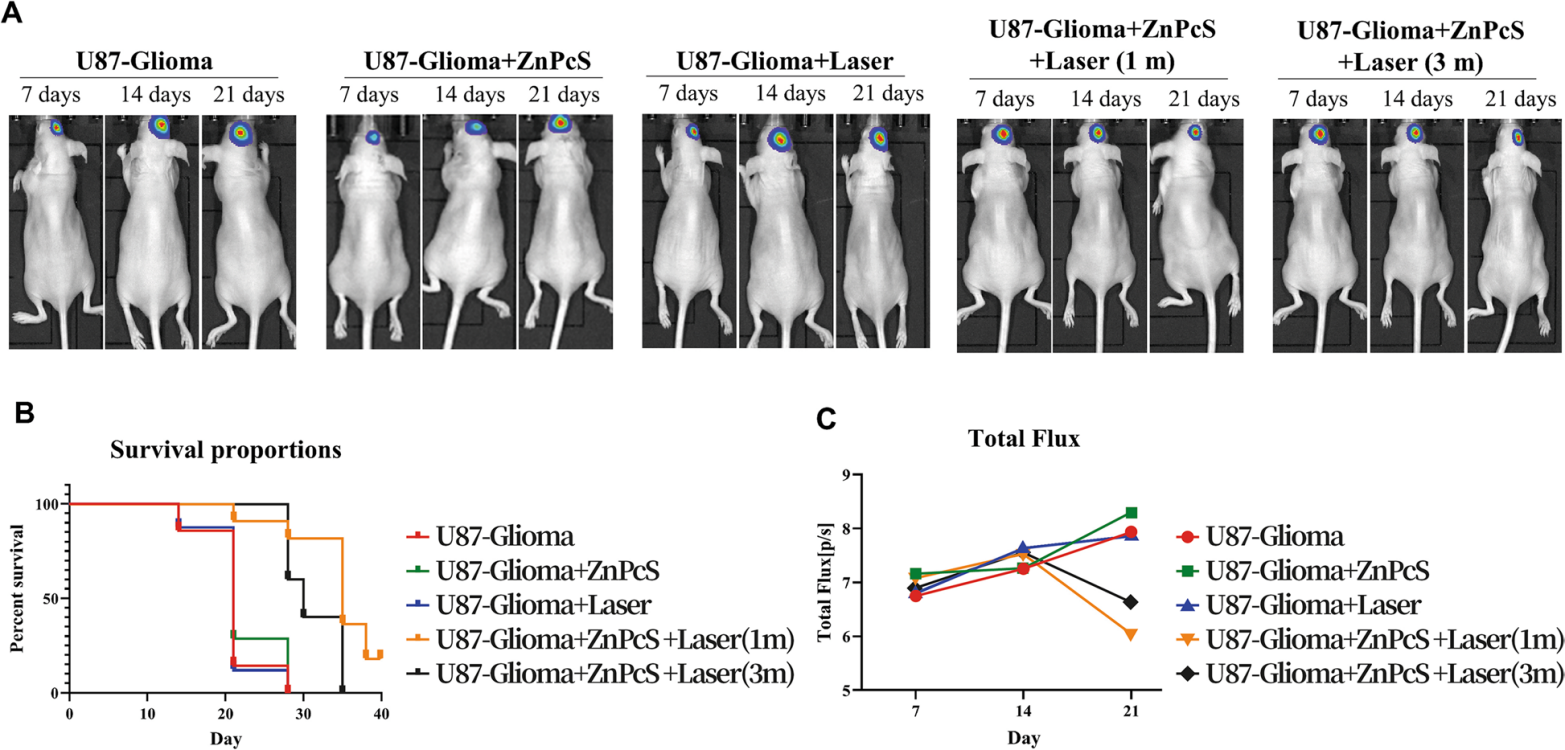
Effect of ZnPcS on U87-glioma transplanted in mice. **a** Tumor growth curves in mice with U87-glioma xenografts after different treatments: glioma only xenografts (nontreatment; *n* = 10), ZnPcS injection without laser irradiation (*n* = 10), non-ZnPcS injection with laser irradiation (*n* = 10), ZnPcS intraperitoneal injection with laser for 1 min (*n* = 10), and ZnPcS intraperitoneal injection with laser for 3 min (*n* = 10). The tumor size is measured using IVIS on day 7, day 14, and day 21 after treatment. **b** The bar graph represents the luciferase intensity expressed by U87-glioma in the mouse brain. Quantitative data are represented as mean ± SEM. **c** Survival Kaplan–Meier curves for ZnPcS and laser-treated mice and control groups

To establish the administration method of ZnPcS in an animal model transplanted with glioma, ZnPcS was injected using a direct injection method with a stereotaxic apparatus and an intraperitoneal injection method. Direct injection to the tumor site using a stereotaxic apparatus is the most effective method for targeting tumors; however, this method has not been considered an injection method for ZnPcS, as it is invasive. In this study, we used the IP method as the most effective non-invasive injection method. After IP injection of ZnPcS, we checked whether ZnPcS reached the brain most effectively at intervals of 30 min, 1 h, 2 h, 4 h, 24 h, 84 h, and 93 h using in vivo imaging of ZnPcS (Supplementary Fig. 2, Additional File 1) and observed that 1 h after ZnPcS injection was the most effective time. When ZnPcS was injected intraperitoneally, ZnPcS appeared to accumulate in the brain over time; however, ZnPcS also appeared to have accumulated in other organs. Therefore, in this study, 1 h after ZnPcS injection was determined to be the most appropriate and effective time. After transplantation of 5 × 10^6^ glioma cells using a stereotaxic apparatus, ZnPcS was intraperitoneally injected one week after mouse stabilization. To activate ZnPcS and trigger ROS production, the mouse brain was irradiated with a laser for 1 and 3 min. Thereafter, the glioma size was measured using IVIS equipment for 7, 14, and 21 days. The transplanted glioma in the mouse brain without ZnPcS and laser irradiation increased in size by approximately 30% after 21 days (Fig. 5A and B). However, in the group irradiated with a laser, the glioma size was reduced by 83.6% (laser irradiation for 1 min) and 75.9% (laser irradiation for 3 min) compared to the tumor group. This effect was observed to increase the average lifespan of mice treated with ZnPcS compared to that of the control tumor group. The Kaplan–Meier survival curve showed that the survival difference between the control tumor group and the ZnPcS-laser-treated group yielded statistically significant results (log-rank *p* < 0.001) (Fig. 5C). The 50% survival rate in the treatment group was 35 days, while that in the control group was 21 days. These results showed that ZnPcS can exert a therapeutic effect by triggering glioma cell death in a non-invasive manner.

**Fig. 6:**
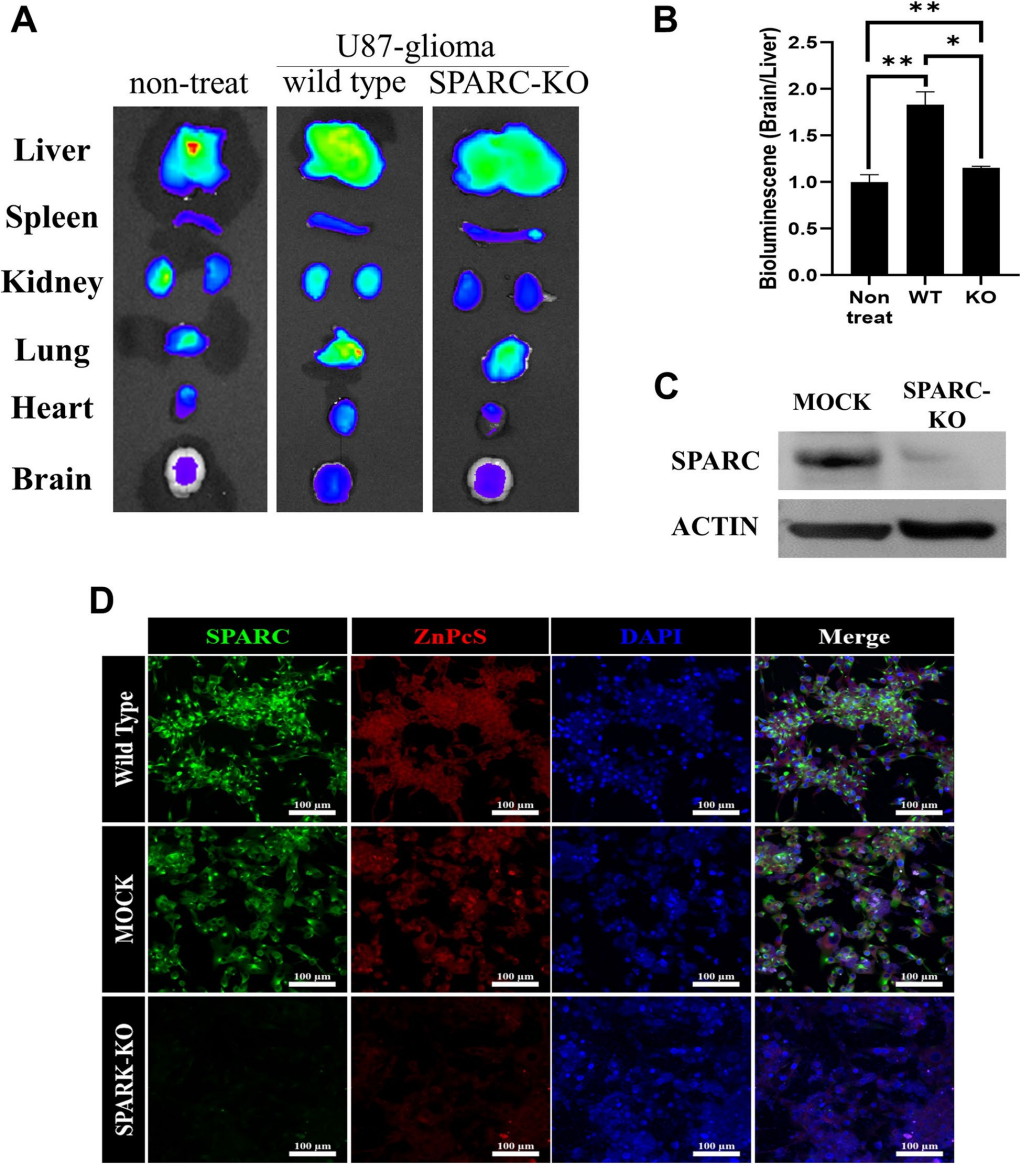
In vivo imaging of ZnPcS distribution in mice bearing U87-glioma (*n* = 4). **a** Ex vivo imaging of the major organs dissected from mice. **b** Ex vivo radiant efficiency of brain tumors. To show how selectively the ZnPcS enters the tumor in the brain, it is marked by dividing the radiant value in the brain into the radiant value in the liver. Quantitative data are represented as the mean ± SEM. Statistical significance is examined by students t-test (*p < 0.05 and **p < 0.01 compared with the nontreated group). **c** Western blot analysis of SPARC protein in the wild type and KO type U87-glioma cells. Actin is used for loading control. **d** Confocal images of SPARC protein and ZnPcS. ZnPcS (red fluorescence) is excited at 633 nm and monitored at 710 nm, SPARK (green fluorescence) is excited at 488 nm and monitored at 516 nm, and nucleus (blue fluorescence) is excited at 405 nm and monitored at 459 nm. Scale bar = 100 μm

Next, a biodistribution experiment was performed to determine whether ZnPcS was selectively introduced into the mouse brain, in which the glioma was expressed. As shown in Fig. 6, after IP injection of ZnPcS, ZnPcS spread throughout the body and accumulated in major organs over time. A particularly important result was that ZnPcS accumulation was significantly increased in the brain of the mouse transplanted with glioma compared to that in the control group (Fig. 6A and B). More importantly, to determine whether ZnPcS can be selectively targeted via SPARC overexpression in the glioma membrane, the selective inflow of ZnPcS was observed using U87-glioma cells that knocked out SPARC protein with CRISPR-Cas9. It was first observed by western blotting that the expression of the SPARC protein was inhibited by CRISPR-Cas9 (Fig. 6C). The immunostaining results showed that SPARC in wild type U87 cells was well expressed, but SPARC expression did not appear in CRISPR-Cas9-treated knock-out (KO) U87 cells (green fluorescence: Fig. 6D). In addition, after ZnPcS treatment, ZnPcS inflow was observed in wild-type U87 cells; however, no inflow of ZnPcS was observed in SPARC-KO cells (Fig. 6D: red fluorescence). As expected, the ZnPcS inflow was significantly decreased in the mouse brains transplanted with glioma cells in which the SPARC gene was knocked out, and it appeared to inflow at the control group level, which was not transplanted with U87 cells (Fig. 6A and B). These results show that ZnPcS can be selectively introduced into gliomas overexpressing SPARC by passing through the BBB. This indicates that ZnPcS-HSA is a mechanism that enables glioma cell death by increasing selective inflow into glioma.

## Discussion

Despite the various treatment approaches, including surgery and chemotherapy, employed for treating glioma—the most common malignant brain tumor—the clinical results are poor [29]. Astrocyte-derived glioma, a type of glial cell, is known to be caused by several genetic mutations; however, the exact cause remains unknown. In addition, this type of tumor is difficult to treat effectively because it easily penetrates other brain tissues [1]. The main cause of treatment failure is that it is difficult to surgically remove glioma that has invaded other brain tissues while preserving brain function; moreover, not only is access to the brain difficult for the developed drug molecule, but there is also a high chance of recurrence due to high resistance [30]. TMZ, a DNA alkylating agent, is clinically used as the most commonly used non-invasive treatment for glioma. However, TMZ has a short plasma half-life (1.8 h), and there is resistance due to long-term treatment, resulting in tumor recurrence in 60–75% of patients treated with TMZ. In addition, high concentrations of TMZ can cause hematological toxicity, acute cardiomyopathy, and hepatotoxicity in patients, resulting in the discontinuation of treatment [31–33]. Therefore, an approach that can pass through the BBB and directly target gliomas to trigger apoptosis is required for effective glioma treatment.

The BBB allows certain small molecules to pass through by passive diffusion, as well as active transport of ions, macromolecules, and nutrients, such as glucose and amino acids, for the functional support of neurons; however, water-soluble molecules require special channels or carrier proteins to move across the BBB [34, 35]. The brain is the most energy-consuming organ in the human body, and it actively absorbs nutrients through the BBB. In particular, absorption of nutrients, such as albumin, is required to support the rapid proliferation of gliomas. Previous studies have suggested the possibility of an important pathway for albumin to pass through the BBB, as SPARC expression was maintained at a relatively low level in normal BBB capillaries, whereas it was upregulated by the tumor-derived secreted factor [17, 36, 37]. The key to this phenomenon is that SPARC is overexpressed in solid cancers, including glioma; thus, it not only triggers tumor growth and angiogenesis, but also increases the absorption of albumin, helping glioma proliferation. Albumin functions as a ligand that can precisely target gliomas and trigger transcytosis across the BBB, enabling efficient targeting and intertumoral drug entry for brain tumor treatment [38, 39]. Therefore, albumin binding was expected to have a significant effect on brain drug delivery in this study.

Among the anticancer drugs devised thus far, light stimulation is known to be ideal for activating drugs and agents to achieve external control (e.g., apoptosis). Typically, photodynamic therapy (PDT) and photothermal therapy are recognized as new approaches in which anti-cancer activity can be triggered by light in the near-infrared region where high levels of tissue penetration occur [40–42]. The advantage of this method is that if agents can be accurately introduced into tumor cells, only cancer cells can be removed without damage to normal cells by light activity alone. In particular, when organs need to be sufficiently preserved during the surgical operation of tumors deep in the brain tissue, such as glioma, light-responsive anticancer systems may be effective in tumor removal.

Toxicity and safety remain major concerns in the development of new anticancer drugs and agents. In fact, to use ZnPcS for glioma treatment, more investigations are required for safety evaluation, including acute toxicity, carcinogenicity, and excretion in vitro. Nevertheless, our findings showed that the risk of cytotoxicity is rather low compared to that of conventional drugs, such as the lack of cytotoxicity to ZnPcS observed in vitro. However, in the case of PDT, which secretes ROS through laser irradiation, it will be important for future research in the development of anticancer drugs and cytotoxicity control to determine how precisely ZnPcS accumulates in the cells and to investigate the conditions for laser treatment. In this study, the accumulation of ZnPcS in glioma was observed at various times after ZnPcS treatment, and ZnPcS accumulation in glioma gradually increased over time after ZnPcS injection. It was also noted that ZnPcS spreads significantly to the surrounding tissues over time; nevertheless, laser irradiation within 1 h after ZnPcS injection into IP could effectively kill glioma while protecting the surrounding tissues.

## Conclusion

In this study, we showed that albumin-binding ZnPcS could induce glioma apoptosis upon laser irradiation. In particular, albumin-binding ZnPcS could enhance BBB penetration, glioma invasion, and cellular uptake in glioma. The overexpression of SPARC provides an uptake pathway for albumin-binding ZnPcS in glioma. Thus, this study developed a new strategy for drug delivery to the brain and a treatment that can overcome the limitations of existing glioma therapies.

## Supplementary Information

**Additional file 1: Synthesis of ZnPcS.** **Supplementary Figure 1.** Cytotoxic effects of U-87 cells incubated with ZnPcS both in the presence and absence of laser. **Supplementary Figure 2.** In-vivo imaging of ZnPcS accumulation in the brain over time.

## Data Availability

All experimental data required to reproduce the findings of this study will be made available to interested investigators.

## Abbreviations

BBB: Blood-brain barrier
SPARC: Secreted protein acidic and rich in cysteine
TMZ: Temozolomide
ZnPcS: A-(6-sulfonatonaphthaleneoxyl) phthalocyaninato zinc
ROS: Reactive oxygen species
DMSO: Dimethyl sulfoxide
DCFH-DA: Dichlorofluorescin diacetate
DBU: 1,8-diazabicyclo-[5.4.0]undec-7-ene
HSA: Human serum albumin
OPLS: Optimized potential for liquid simulation
ATCC: American Type Culture Collection
FBS: Fetal bovine serum
HUVEC: Human umbilical vein endothelial cell
BSA: Bovine serum albumin
RMSDs: Root-mean-square deviations
DCF-DA: 2,7-dichlorofluorescin diacetate
KO: Knock-out
PDT: Photodynamic therapy
