# Supplementary material for: Albumin-binding photosensitizer capable of targeting glioma via the SPARC pathway
Source: Biomater Res. 2023 Mar 21;27:23. doi: 10.1186/s40824-023-00360-3 (PMC10031904; doi:10.1186/s40824-023-00360-3)
Supplement: Supplementary file 1 — Additional file 1: Synthesis of ZnPcS. Supplementary Figure 1. Cytotoxic effects of U-87 cells incubated with ZnPcS both in the presence and absence of laser. Supplementary Figure 2. In-vivo imaging of ZnPcS accumulation in the brain over time. [file 40824_2023_360_MOESM1_ESM.docx]

**Supplementary information**

**Albumin-binding photosensitizer capable of targeting glioma via the SPARC pathway**

Xingshu Li^a,#^, Jae Sang Oh^b,#^, Yoonji Lee^c,#^, Eun Chae Lee^b^, Mengyao Yang^d^, Nahyun Kwon^d^, Tae Won Ha^e^, Dong-Yong Hong^2^, Yena Song^e^, Hyun Kyu Kim^e^, Byung Hoo Song^e^, Sun Choi ^f,^*, Man Ryul Lee^e,^* and Juyoung Yoon^d,^*

a. Fujian Provincial Key Laboratory for Cancer Metastasis Chemoprevention and Chemotherapy, College of Chemistry, Fuzhou University, China

b. Department of Neurosurgery, College of Medicine, Soonchunhyang University, Cheonan Hospital, Republic of Korea

c. College of Pharmacy, Chung-Ang University, Republic of Korea.

d. Department of Chemistry and Nanoscience, Ewha Womans University, Republic of Korea.

e. Soonchunhyang Institute of Medi-bio Science (SIMS), Soonchunhyang University, Republic of Korea

f. Global AI Drug Discovery Center, College of Pharmacy and Graduate School of Pharmaceutical Sciences, Ewha Womans University, Republic of Korea.

# These authors contributed equally to this work.

* Corresponding authors: Tel: +82-2-3277-4503; Fax: +82-2-3277-2851; e-mail: sunchoi@ewha.ac.kr (S.C.); Tel: +82-2-3277-2400; Fax: +82-2-3277-2385; e-mail: jyoon@ewha.ac.kr (J.Y.); Tel: +82-41-413-5013; Fax: +82-41-413-5006; e-mail: leeman@sch.ac.kr (M.R.L.).

**Synthesis of ZnPcS**

A mixture of 3-nitrophthalonitrile (1.73 g, 10 mmol), potassium 6-hydroxy-2-naphthalenesulfonate (2.62 g, 10 mmol), and anhydrous K_2_CO_3_ (2.76 g, 20 mmol) in DMSO (20 mL) was stirred at 45 °C for 48 h under an atmosphere of nitrogen. The reaction mixture was filtrated and the filtrate was poured into chloroform. The resulting yellow solid was filtered and washed with chloroform, ethanol, and acetone, and then dried in vacuo. The product was afforded as a light-yellow solid (3.7 g, 95.2 %). ^1^H NMR (300 MHz, DMSO-d_6_): δ = 8.23 (s, 1 H, Ar-H), 8.17 (d, J = 9.3 Hz, 1 H, Ar-H), 7.82-7.91 (m, 3 H, Ar-H), 7.79-7.72 (m, 2 H, Ar-H), 7.48 (dd, J = 2.4, 9.0 Hz, 1 H, Ar-H), 7.41 ppm (dd, J = 1.5, 8.4 Hz, 1 H, Ar-H). HRMS (ESI): m/z Calcd for C_18_H_9_N_2_O_4_S [M-K]^-^ 349.0289, found 349.0292.

A mixture of 3-[6-(potassium sulfonate)naphthaleneoxyl] phthalonitrile (1.52 g, 3.0 mmol), phthalonitrile (1.92 g, 15.0 mmol), and anhydrous K_2_CO_3_ (0.4 g, 3.0 mmol) in n-pentanol (40 mL) was stirred at 90 °C under an atmosphere of nitrogen for 30 min; zinc acetate (1.8 g, 9.8 mmol) and 1,8-diazabicyclo-[5.4.0]undec-7-ene (DBU) (1.8 mL, 12.1 mmol) were then added. The resulting mixture was stirred at 140 °C for 48 h. After being cooled, the reaction mixture was filtrated, and the residue was washed by DMF. After collecting the filtrate and concentrating, the crude product was first purified by silica gel column chromatography using CH_2_Cl_2_ and EA/DMF (1/1) as eluent to obtain a green/blue crude. The mixture was concentrated under reduced pressure and purified by size exclusion chromatography on a Bio-Beads S-X3 column using DMF as eluent. The crude product was further purified by recrystallization from a mixture of DMF and chloroform to afford a green/blue solid (0.32 g, 12.7%). ^1^H NMR (300 MHz, DMSO-d_6_): δ = 9.19-9.23 (m, 6 H, Pc-α-H), 8.76 (d, J = 7.8 Hz, 1 H, Pc-α-H), 8.17-8.26 (m, 8 H, Pc-β-H), 8.13-8.17 (m, 1 H, Ar-H), 7.91-7.96 (m, 1 H, Ar-H), 7.56-7.69 (m, 2 H, Ar-H), 7.54 (d, J = 1.8 Hz, 2 H, Ar-H). HRMS (ESI): m/z Calcd for C_42_H_21_N_8_O_4_SZn [M-K]^-^ 797.0703, found 797.0709.

**Supplementary figures**

**
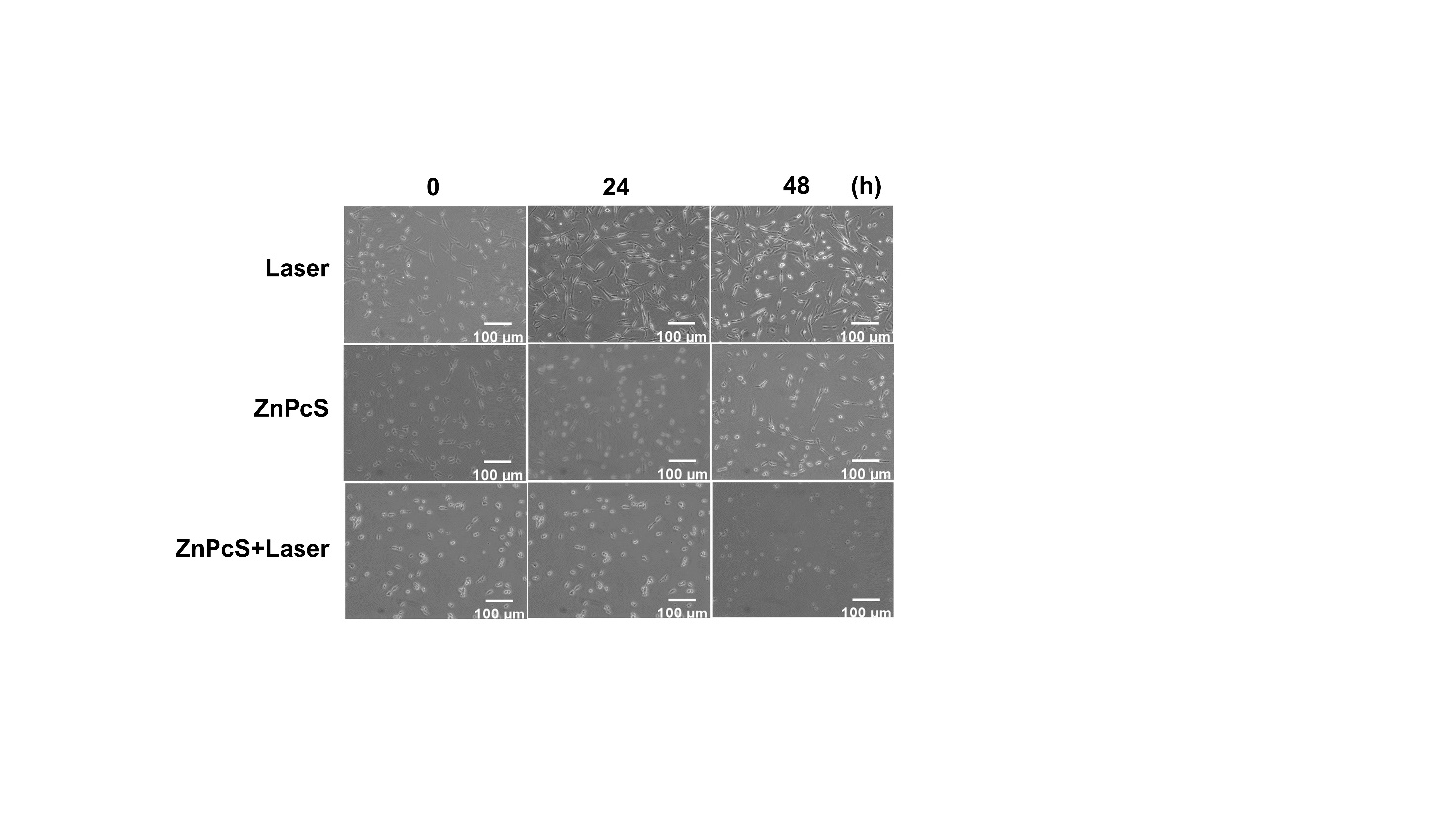
**

**Supplementary Figure 1.** Confluency and cell death were visually assessed by phase contrast microscopy. Cytotoxic effects of U-87 cells incubated with ZnPcS both in the presence and absence of laser.


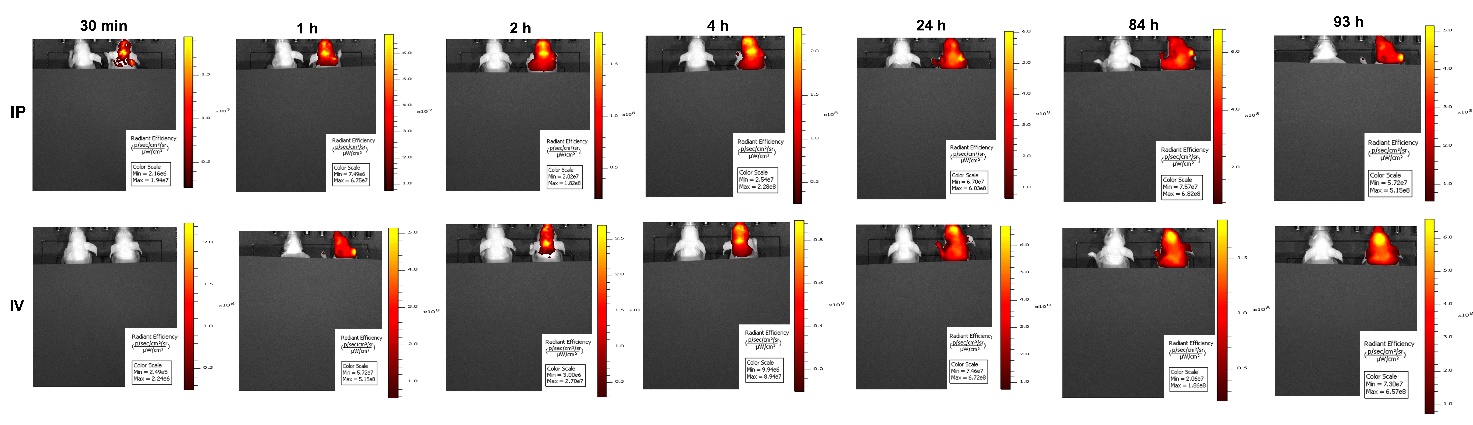


**Supplementary Figure 2.** In-vivo imaging of ZnPcS accumulation in the brain over time.
